# G9a co-suppresses LINE1 elements in spermatogonia

**DOI:** 10.1186/1756-8935-7-24

**Published:** 2014-09-11

**Authors:** Monica Di Giacomo, Stefano Comazzetto, Srihari C Sampath, Srinath C Sampath, Dónal O’Carroll

**Affiliations:** 1 European Molecular Biology Laboratory (EMBL), Mouse Biology Unit, Via Ramarini 32, Monterotondo Scalo 00015, Italy; 2 Genetics Department, Genomics Institute of the Novartis Research Foundation, 10675 John Jay Hopkins Drive, San Diego, CA 92121, USA

**Keywords:** LINE1, Retrotransposons, IAP, G9a, H3K9me2, piRNA and DNA methylation

## Abstract

**Background:**

Repression of retrotransposons is essential for genome integrity and the development of germ cells. Among retrotransposons, the establishment of CpG DNA methylation and epigenetic silencing of LINE1 (L1) elements and the intracisternal A particle (IAP) endogenous retrovirus (ERV) is dependent upon the piRNA pathway during embryonic germ cell reprogramming. Furthermore, the Piwi protein Mili, guided by piRNAs, cleaves expressed L1 transcripts to post-transcriptionally enforce L1 silencing in meiotic cells. The loss of both DNA methylation and the Mili piRNA pathway does not affect L1 silencing in the mitotic spermatogonia where histone H3 lysine 9 dimethylation (H3K9me2) is postulated to co-repress these elements.

**Results:**

Here we show that the histone H3 lysine 9 dimethyltransferase G9a co-suppresses L1 elements in spermatogonia. In the absence of both a functional piRNA pathway and L1 DNA methylation, G9a is both essential and sufficient to silence L1 elements. In contrast, H3K9me2 alone is insufficient to maintain IAP silencing in spermatogonia. The loss of all three repressive mechanisms has a major impact on spermatogonial populations inclusive of spermatogonial stem cells, with the loss of all germ cells observed in a high portion of seminiferous tubules.

**Conclusions:**

Our study identifies G9a-mediated H3K9me2 as a novel and important L1 repressive mechanism in the germ line. We also demonstrate fundamental differences in the requirements for the maintenance of L1 and IAP silencing during adult spermatogenesis, where H3K9me2 is sufficient to maintain L1 but not IAP silencing. Finally, we demonstrate that repression of retrotransposon activation in spermatogonia is important for the survival of this population and testicular homeostasis.

## Background

The L1 element is the most successful mobile genetic element in mammalian genomes [1]. Processes that ensure L1 silencing are of paramount importance to germ cell development and ultimately the quality of gametes. Spermatogonia are the mitotic germ cells of the testis comprising of the spermatogonial stem cells as well as a pool of transit-amplifying cells that support gamete formation throughout adult life. CpG promoter DNA methylation and the post-translational Piwi-interacting RNA (piRNA) pathway have proven roles in the maintenance of L1 silencing during adult spermatogenesis [2,3]. DNA methylation of L1 promoter elements epigenetically represses L1 transcription in both somatic and germ cells [2,4]. In parallel, piRNAs complementary to L1 sequences guide the Piwi proteins Mili or Miwi to cleave and destroy expressed L1 transcripts via an RNA interference-like mechanism [3,5,6]. In addition, the Piwi proteins Mili and Miwi2 direct *de novo* DNA methylation of L1 and IAP elements during embryonic germ cell development [5,7-9]. In *Mili*^*-/-*^ mice, both DNA methylation and the Piwi-piRNA pathways are lost, with L1 deregulation observed only at the onset of pachytene in meiosis [3]. Thus, additional mechanisms must exist over and above DNA methylation and the Piwi-piRNA pathway that repress L1 in spermatogonia. The repressive H3K9me2 histone modification has been shown to be present in spermatogonia, resident across L1 elements in lepto/zygotene cells, and lost when cells reach pachytene [3,10]. However, the importance of this potential repressive mechanism in the maintenance of L1 silencing remains unknown. Here, we sought to address the physiological significance of H3K9me2 in L1 repression and spermatogonial populations.

## Results and discussion

**Figure 1:**
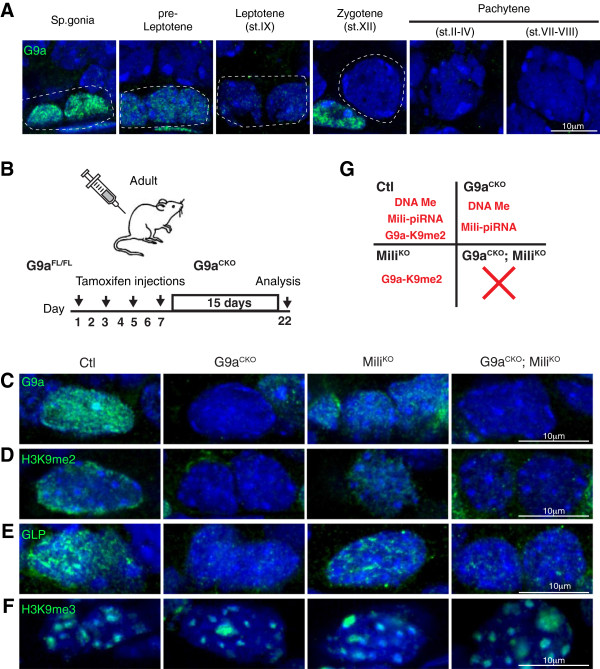
**Expression and conditional ablation of G9a in the adult testis. (A)** Immunofluorescences using anti-G9a antibody on wild type germ cells from adult testis sections are shown. Dashed lines outline the indicated cell type. **(B)** Overview of deletion protocol for inducible G9a ablation and analysis. **(C-F)** Immunofluorescences using anti-G9a **(C)**, anti-H3K9me2 **(D)**, anti-GLP **(E)** and anti-H3K9me3 **(F)** antibodies on spermatogonia from testis sections of the indicated genotypes are shown. **(G)** Scheme indicating the repressive L1 mechanisms functioning in spermatogonia of the respective genotypes.

The G9a HMTase complex is responsible for the bulk of euchromatic H3K9me2 in numerous cells types [10-14]. We found abundant G9a expression in spermatogonia, a reduction in preleptotene cells and absence in subsequent stages of spermatogenesis (Figure 1A). Therefore the loss of G9a precedes the complete loss of H3K9me2, thus identifying G9a as an excellent candidate for the deposition of the L1-resident H3K9me2. The conditional ablation of G9a during embryonic germ cell development results in meiotic arrest during adult spermatogenesis without L1 deregulation [10]. To understand if G9a-mediated H3K9me2 co-suppresses L1 along with DNA methylation and the Mili-piRNA pathway, we induced G9a deficiency in the Mili null background. To this end we combined the *G9a*^*Fl*^, *Mili*^*-*^ and the tamoxifen (TMX)-inducible *Rosa26*^*ERT2Cre*^ alleles. To induce G9a deletion, TMX was administered every second day over eight days in adult mice (Figure 1B). The experimental cohort consisted of *R26*^*ERT2Cre/+*^, *Mili*^*-/-*^*; R26*^*ERT2Cre/+*^, *G9a*^*Fl/Fl*^*; R26*^*ERT2Cre/+*^ and *G9a*^*Fl/Fl*^*; Mili*^*-/-*^*; R26*^*ERT2Cre/+*^ mice that upon TMX treatment become our control (Ctl), Mili^KO^, G9a^CKO^ and G9a^CKO^; Mili^KO^ mice, respectively. This protocol resulted in the conditional ablation of G9a, accompanied by the entire loss of H3K9me2 (Figure 1C-D). Furthermore, the deletion of G9a resulted in the loss of its associated partner HMTase G9a-like protein (GLP) (Figure 1E) as has been previously described in other systems [13,15,16]. Finally, the loss of G9a-mediated H3K9me2 did not affect H3K9me3 in spermatogonia that retain the appropriate constitutive heterochromatin staining (Figure 1F), which is similar to what has been reported *in vivo* in G9a-deficient meiotic [10] or neuronal cells [13]. Thus, in our experiments, G9a^CKO^ mice lose G9a-GLP-mediated H3K9me2, L1 CpG DNA methylation and piRNA pathway are both lost in Mili^KO^ animals, whereas all three repressive mechanisms are absent in the G9a^CKO^; Mili^KO^ mice (Figure 1G).

**Figure 2:**
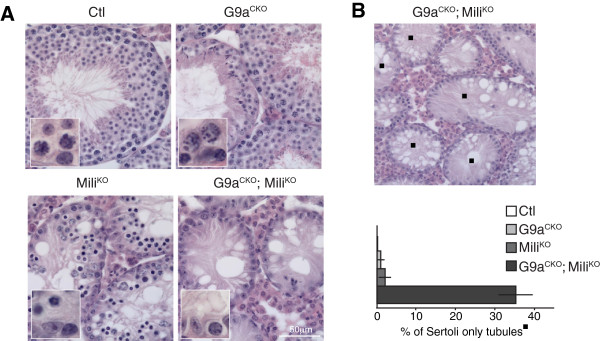
**Induced loss of G9a in Mili**^**-/- **^**mice results in severe spermatogenic defects. (A)** Hematoxylin and eosin stained adult testis sections from the indicated genotypes. The inset highlights the basal portion of the tubule containing spermatogonia and meiotic cells or spermatagonia only in the case of G9a^CKO^; Mili^KO^ mice. **(B)** Representative image of G9a^CKO^; Mili^KO^ hematoxylin and eosin stained adult testis section, the black square indicates Sertoli-only tubules. The percentage of Sertoli cell-only tubules in the respective genotypes is shown. The results are derived from four mice of the indicated genotypes and the s.e.m is shown.

This induced G9a-deficiency in adult testis resulted in majorly disrupted spermatogenesis. Abnormal seminiferous tubules were observed with a significant reduction in meiotic cells and the presence of round spermatids is a likely remnant of a spermatogenic wave prior to the induced G9a deletion (Figure 2A). The disruption of Mili resulted in a pachytene arrest, accompanied with L1 derepression [3,9,17] (Figure 2A). However the induced ablation of G9a in the background of Mili-deficiency in G9a^CKO^; Mili^KO^ mice had profound consequences on the seminiferous tubules beyond those seen in the individual gene disruptions. First, in a subset and in the majority of tubules, spermatogonia were the only germ cells remaining (Figure 2A). Thus, the combined loss of both G9a and Mili resulted in the elimination of all meiotic cells. The second subset, constituting approximately 35% of the tubules, contained only the somatic Sertoli cells with the complete loss of all germ cells (Figure 2A-B). This phenotype indicates the loss of the stem cell compartment within these tubules. In summary, the conditional loss of G9a in the background of Mili-deficiency had a profound impact on the seminiferous tubules eliminating all meiotic cells, as well as affecting spermatogonia inclusive of spermatogonial stem cells.

**Figure 3:**
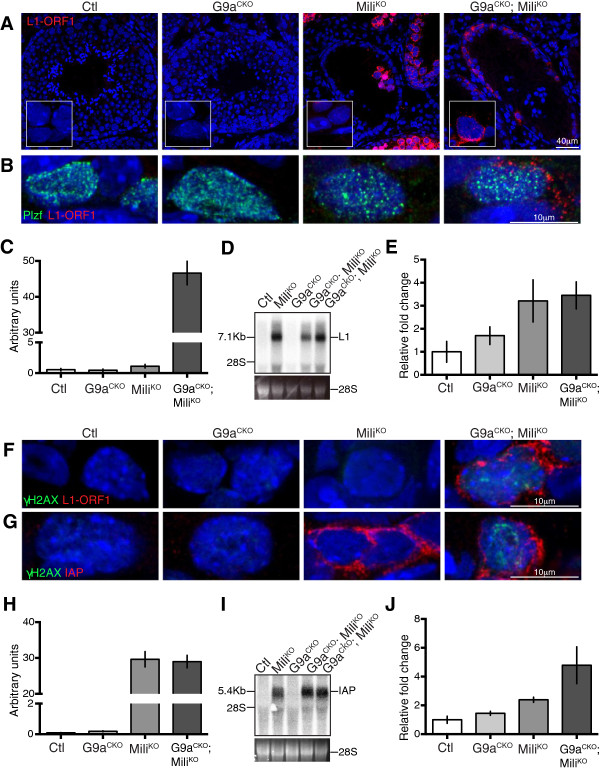
**G9a co-suppresses L1 in spermatogonia. (A)** Immunofluorescence using anti-L1 ORF1 antibody on adult testis sections of the indicated genotypes are shown. The inset highlights a crop section containing spermatogonia. **(B)** Immunofluorescence using anti-L1 Orf1 and anti-PLZF antibodies on spermatogonia from testis sections of the indicated genotypes are shown. **(C)** Quantification of L1 Orf1 signal from immunofluorescence of spermatogonia cells of the indicated genotype is shown. Bars represent mean ± s.e.m. (n = 20 to 40 cells). **(D)** Northern blot containing testicular RNA of the indicate genotypes probed with an L1 probe is shown. Full-length L1 transcripts are indicated. 28S RNA is shown as a loading control. The two G9a^CKO^;Mili^KO^ samples represent biological replicates. **(E)** qRT-PCR measurement of L1 transcripts from testicular RNA of the indicated genotypes is shown. Bars represent mean ± s.e.m. (n = 3). **(F-G)** Immunofluorescence using anti-L1 ORF1 **(F)**, anti-IAP **(G)** and anti-γH2AX antibodies on spermatogonia from testis sections of the indicated genotypes are shown. **(H)** Quantification of IAP Gag protein signal from immunofluorescence of spermatogonia cells of the indicated genotype is shown. Bars represent mean ± s.e.m. (n = 20 to 40 cells). **(I)** Northern blot containing testicular RNA of the indicate genotypes probed with an IAP probe is shown. Full-length IAP transcripts are indicated. 28S RNA is shown as a loading control. The two G9a^CKO^; Mili^KO^ samples represent biological replicates. **(J)** qRT-PCR measurement of IAP transcripts from testicular RNA of the indicated genotypes is shown. Bars represent mean ± s.e.m. (n = 3).

**Figure 4:**
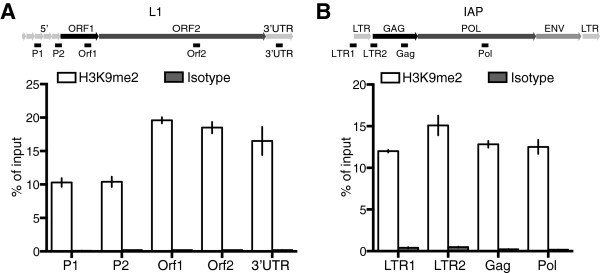
**H3K9me2 resides on L1 and IAP elements in SSCs. (A-B)** qPCR results from chromatin immunoprecipitation using an anti-H3K9me2 (black) and isotype control (grey) antibodies from spermatogonial stem cell lines. A schematic image describing L1 and IAP elements and the position of the primer sets used is depicted. Bars represent mean ± s.e.m. measured in triplicate from a representative experiment performed in two biological replicates.

Next, we analyzed the status of L1 repression through detection of protein encoded by L1 open reading frame 1 (L1 ORF1). As expected L1 ORF1 protein was not detected in spermatogonia or any other germ cell population in G9a^CKO^ mice (Figure 3A). L1 ORF1 was detected within Mili^KO^ tubules but specifically in the meiotic cells [3]. In G9a^CKO^; Mili^KO^ mice L1 Orf1 was detected in spermatogonia within the seminiferous tubules (Figure 3A-C). The identity of these L1 ORF1-expressing cells was confirmed using the undifferentiated spermatogonia marker Plzf (Figure 3B) [18-20]. In the G9a^CKO^; Mili^KO^ testis northern blotting revealed the expression of full-length L1 transcripts that likely constitute intermediates competent for transposition (Figure 3D). This L1 activation was additionally confirmed by qRT-PCR (Figure 3E). Given the loss of meiotic cells in G9a^CKO^; Mili^KO^ mice, the detection of the full-length L1 transcripts must originate from the spermatogonia. Finally, DNA damage as evidenced by γH2AX was detected within the remaining L1 ORF1-expressing G9a^CKO^; Mili^KO^ spermatogonia (Figure 3F). We next analyzed the expression of the endogenous retroviruses IAP and MuERV-L, whose repression in the germ line is dependent and independent of the piRNA pathway, respectively. We could not detect the expression of MuERV-L in any of the genotypes (data not shown). Interestingly, in contrast to L1, the loss of Mili alone was sufficient to deregulate IAP in spermatogonia (Figure 3G-J); however, this activation was not associated with the occurrence of DNA damage (Figure 3G). The above data indicated that H3K9me2 is required to co-suppress L1 but insufficient for IAP silencing, should this mark reside on the respective elements in spermatogonia. To address this question, we took advantage of SSC lines that are derived from neonatal testis and representative of undifferentiated spermatogonia with *in vivo* reconstitution capacity [21]. Enrichment for H3K9me2 in SSCs was observed across both L1 and IAP elements (Figure 4A-B). Therefore, the induced loss of G9a/H3K9me2 in the absence of both L1 DNA methylation and the piRNA pathway results in L1 derepression within spermatogonia.

## Conclusions

Here we demonstrate a role for G9a and H3K9me2 in the repression of L1 elements during adult spermatogenesis. This function of G9a is redundant with CpG DNA methylation and the Mili-piRNA pathway; it is only when all three mechanisms are ablated that L1 becomes derepressed within spermatogonia. In the absence of both DNA methylation and the Mili piRNA pathway, G9a is both necessary and sufficient to maintain L1 silencing. Thus, with respect to other adult germ cell populations, spermatogonia are unique in employing three distinct mechanisms to repress L1. The SSC that maintains spermatogenesis throughout adult life resides within the population of undifferentiated spermatogonia. Therefore having three distinct L1 defensive mechanisms in place within SSCs has major protective advantages for the long-term genomic quality of the gametes. The consequence of L1 activation in spermatogonia are dire as evidenced in the G9a^CKO^; Mili^KO^ mice with either the complete loss of all germ cells, or with spermatogonia being the only germ cells present within the seminiferous tubules. Thus, not only are meiotic cells acutely sensitive to L1 reactivation and the ensuing DNA damage but spermatogonia are as well. The observed genomic damage in G9a^CKO^; Mili^KO^ spermatogonia could be a result of L1 derepression alone or in conjunction with the observed IAP deregulation. The fact that IAP reactivation is observed in Mili^KO^ spermatogonia without DNA damage could indicate that other post-transcriptional mechanism (s) are in place to inhibit IAP translocation such as the APOBEC RNA editing pathway that has been shown to restrict ERVs inclusive of IAP elements [22-24]. Alternatively, the level of IAP translocation is low in Mili^KO^ spermatogonia and does not elicit a robust DNA damage response. It is very interesting that both L1 and IAP retrotransposons that depend upon the piRNA pathway for the establishment of epigenetic silencing [5,7-9] have fundamentally differential requirements for maintenance of their silencing in spermatogonial and meiotic cells. For both L1 and IAP the three mechanisms are in place within spermatogonia, however the derepression of IAP in Mili^KO^ spermatogonia indicates that DNA methylation and or the piRNA pathway are predominantly required for the maintenance of IAP silencing therein. In contrast to L1, the conditional loss of Mili or specifically its endonuclease activity in meiotic cells that does not affect CpG DNA methylation patterns, has no impact on IAP silencing [3]. As H3K9me2 is globally lost in pachytene spermatocytes [3], IAP repression in Mili^CKO^ spermatocytes that are devoid of the piRNA post-transcriptional silencing pathway would indicate that CpG DNA methylation is sufficient and the key mechanism for the maintenance of IAP silencing. In summary, here we show that G9a-mediated H3K9me2 is sufficient to maintain L1 silencing in the absence of L1 CpG DNA methylation and the piRNA pathway within spermatogonia. Finally, as G9a is broadly expressed our findings may indicate a role for G9a and H3K9me2 in L1 silencing in other cell types.

## Methods

### Mouse strains

The *Mili*^*-*^, *G9a*^*Fl*^ and *Rosa26*^*ERT2Cre*^ alleles were described previously [3,15,25]. TMX (Sigma) was injected intraperitoneally (i.p.) at a concentration of 75 mg/kg in corn oil (Sigma) as described in the text. All mice were analyzed 15 days after the last TMX injection. All mouse breeding and experimentation was performed in the EMBL Mouse Biology Unit, Monterotondo with ethical approval from the EMBL Animal Welfare and Ethical Review Body and in accordance with current Italian legislation (Art. 9, 27. Jan 1992, n^o^116) under license from the Italian health ministry. Requests for G9a^Fl^ mice should be addressed to Alexander Tarakhovsky (The Rockefeller University). The Mili^-^ allele is available from European Mouse Mutant Archive (https://www.infrafrontier.eu/infrafrontier-research-infrastructure/international-collaborations-and-projects/european-mouse) on a non-collaborative basis.

### Antibodies, immunofluorescence and histology

Rabbit polyclonal L1 ORF1 antisera were made through immunization of rabbits with recombinant ORF1 protein. The following antibodies were used at the indicated dilutions for IF: anti-G9a (1:50) (A. Tarakhovsky, The Rockefeller University, New York, NY, USA), anti-ORF1 L1 (1:500), mouse monoclonal anti-GLP (R&D systems, Minneapolis, MN, USA, PP-B0422-00) (1:100), anti-IAP Gag (1:500) (B. Cullen, Duke University, Durham, NC, USA), mouse monoclonal anti-γH2AX (Abcam, Cambridge, UK ab26350) (1:500), mouse monoclonal anti-H3K9me2 (Abcam, Cambridge, UK ab1220) (1:100) and rabbit polyclonal anti-Plzf (Santa Cruz, Dallas, TX, USA sc-22839) (1:100). Immunofluorescence and histology were performed as described [3]. Quantification of L1 Orf1 and IAP signal from at least 20 to 40 cells was performed using Fiji software (http://fiji.sc/Fiji).

### Northern blotting

Northern blotting detection of L1, IAP and MuERV-L transcripts from total testicular RNA was performed as described [9] using an L1Md-A2 [3], IAP [9] and MuERV-L probe [26].

### Quantitative PCR

Quantitative PCR from total testis was performed as previously described [5]. H2Afz was used as a loading control between samples. For MuERV-L and H2Afz detection, the primers used were as follows: MuERV-L-FW 5′- CACAGCTGCGACTGAACAAT -3′; MuERV-L-RV 5′- CTAGAACCACTCCTGGTACCAAC -3′; H2Afz-FW 5′-ACAGCGCAGCCATCCTGGAGTA-3′; H2Afz-RV 5′-TTCCCGATCAGCGATTTGTGGA-3′.

### Chromatin immunoprecipitation assay

Chromatin immunoprecipitation with both H3K9me2 and isotype control antibodies were performed as previously described [3]. Briefly, *in vitro* cultured SSC cells [21] were FACS-sorted from primary mouse embryonic fibroblast feeder layer and fixed for 10 minutes in 4% formaldehyde. Three millions cells were used as input for every CHIP experiment. Quantitative PCR on the immunoprecipitated DNA was performed using L1 primers as previously described [3]. For IAP, primers were as follows: LTR1-FW 5′-TGGTAAACAAATAATCTGCGCATGA-3′; LTR1-RV 5′-CACTCCCTGATTGGCTGCAG-3′; LTR2-FW 5′-GTGAGAACGCGTCGAATAACAAT-3′; LTR2-RV 5′- GTGATCCGTAGTTCTGGTTCTGA-3′; Gag-FW 5′-GGACTCTTACTCTAGCTGCTAACC-3′; Gag-RV 5′-AAGACACACAAACTGAAAGGCTG-3′; Pol-FW 5′- TAATGTCCCTCGTCTTGGTGATG-3′; Pol-RV 5′-ATACATCACCGTCATTGGGAGTG-3′.

## Abbreviations

CKO: conditional knock out; ERV: endogenous retrovirus; HMTase: histone methyltransferase; H3K9me2: histone H3 lysine 9 dimethylation; IAP: intracisternal A particle; i.p.: intraperitoneally; KO: knock-out; LINE1 or L1: long interspersed elements 1; MuERV-L: murine endogenous retrovirus-like; ORF1: open reading frame 1; piRNA: Piwi-interacting RNA; qRT-PCR: quantitative reverse-transcriptase PCR; SSC: spermatogonial stem cell; TMX: tamoxifen.

## Competing interests

The authors declare that they have no competing interests.

## Authors’ contributions

MDG designed and performed the majority of the experiments. SC performed the northern blotting, ChIP and qRT-PCR experiments. Srihari CS provided the G9a^Fl^ allele. Srinath CS generated the G9a antibody. DO’C conceived and supervised the experiments and wrote the manuscript. All authors read and approved the final manuscript.
